# Impact of NGO Training and Support Intervention on Diarrhoea Management Practices in a Rural Community of Bangladesh: An Uncontrolled, Single-Arm Trial

**DOI:** 10.1371/journal.pone.0112308

**Published:** 2014-11-14

**Authors:** Ahmed S. Rahman, Mohammad Rafiqul Islam, Tracey P. Koehlmoos, Mohammad Jyoti Raihan, Mohammad Mehedi Hasan, Tahmeed Ahmed, Charles P. Larson

**Affiliations:** 1 Centre for Nutrition and Food Security, International Centre for Diarrhoeal Diseases Research, Bangladesh (icddr,b), Dhaka, Bangladesh; 2 CCEB, School of Medicine and Public Health, University of Newcastle, Newcastle, Australia; 3 Department of Health Administration and Policy, College of Health and Human Services, George Mason University, Fairfax, Virginia, United States of America; 4 Department of Pediatrics, University of British Columbia and Centre for International Child Health, BC Children's Hospital, Vancouver, British Columbia, Canada; Fondazione IRCCS Ca' Granda Ospedale Maggiore Policlinico, Università degli Studi di Milano, Italy

## Abstract

**Purpose/Objective:**

The evolving Non-Governmental Organization (NGO) sector in Bangladesh provides health services directly, however some NGOs indirectly provide services by working with unlicensed providers. The primary objective of this study was to examine the impact of NGO training of unlicensed providers on diarrhoea management and the scale up of zinc treatment in rural populations.

**Methods:**

An uncontrolled, single-arm trial for a training and support intervention on diarrhoea outcomes was employed in a rural sub-district of Bangladesh during 2008. Two local NGOs and their catchment populations were chosen for the study. The intervention included training of unlicensed health care providers in the management of acute childhood diarrhoea, particularly emphasizing zinc treatment. In addition, community-based promotion of zinc treatment was carried out. Baseline and endline ecologic surveys were carried out in intervention and control villages to document changes in treatments received for diarrhoea in under-five children.

**Results:**

Among surveyed household with an active or recent acute childhood diarrhoea episode, 69% sought help from a health provider. Among these, 62.8% visited an unlicensed private provider. At baseline, 23.9% vs. 22% of control and intervention group children with diarrhoea had received zinc of any type. At endline (6 months later) this had changed to 15.3% vs. 30.2%, respectively. The change in zinc coverage was significantly higher in the intervention villages (p<0.01). Adherence with giving zinc for 10 days or more was significantly higher in the intervention households (9.2% vs. 2.5%; p<0.01). Child's age, duration of diarrhoea, type of diarrhoea, parental year of schooling as well as oral rehydration solution (ORS) and antibiotic usage were significant predictors of zinc usage.

**Conclusion:**

Training of unlicensed healthcare providers through NGOs increased zinc coverage in the diarrhoea management of under-five children in rural Bangladesh households.

**Trial Registration:**

ClinicalTrials.gov NCT02143921

## Introduction

Even a decade after into the 21^st^ century and the proven efficacy of oral rehydration salts and zinc therapy, diarrhoea still remains the second leading cause of mortality among under-five children globally [1]–[4]. It has been estimated that diarrhoea has accounted for about 800,000 out of the total 7.6 million under-five child death globally with the highest contribution from the developing countries [5]. The comparison of 69,000 child death attributed to diarrhoea in Bangladesh in a year [6] to the US child mortality of 300 [7], portrays the ravaging effect of diarrhoea in developing countries. A recent estimate has shown that diarrhoea is responsible for 22% of all under-five child deaths in Africa and 23% in South Asia [6]. Therefore, ‘one out of every nine under-5 child death is due to diarrhoea’, is a statement bold enough to illustrate the impact of diarrhoea on child mortality. Despite the huge burden of diarrhoea among under-five children, most do not receive appropriate treatment [8]. WHO diarrhoeal management guidelines include treating patients with hypo-osmolar oral rehydrating salt (ORS) and zinc along with recommendation to avoid unnecessary use of pharmacological agents. Continued feeding has also been suggested [9]. Meta-analyses of several randomized controlled trials have confirmed the preventive and prophylactic effects of zinc on diarrhoeal episodes among under-five children [10]–[12], which complements the WHO guidelines on zinc usage for diarrhoea. It has been estimated that globally, the successful scaling up of zinc treatment for childhood diarrhoea could potentially save 400,000 under-five deaths per year [13]. Antibiotics, on other hand, is indicated in certain types of diarrhoea such as confirmed or suspected cholera, invasive diarrhoea caused by Shigella and E coli. and dysentery induced by other organisms such as Campylobacter spp. [14]. The European Society for Paediatric Gastroenterology, Hepatology and Nutrition (ESPGHAN) has suggested nine components for good diarrhoeal management, which includes, use of oral rehydration solution, use of hypotonic solution (Na 60 mmol/L, glucose 74–111 mmol/L), fast oral rehydration over of the period of 3 to 4 hours followed by normal feeding, avoid using special formula unless justified, avoid using diluted formula unless justified, continued breastfeeding all the time, supplementation with oral rehydration solution and avoid using unnecessary medication [15]. The guideline also stated that more than 8 episodes/day with substantial stool volumes, persistent vomiting, severe underlying disease and age less than 2 months as indications for medical visits for diarrhoeal patients, whereas, telephone consultation is recommended for uncomplicated diarrhoea. As for hospitalization criteria, ESPGHAN recommended the presence of at least one of the following abnormality/condition: shock, severe dehydration (>9% body weight), any neurological abnormalities, intractable or bilious vomiting, ORS treatment failure, failure of caregivers to provide adequate care at home and/or concerns on social/logistic scenario and suspected surgical condition [16]. It is needed to be pointed out that ESPGHAN recommendation for diarrhoeal management does not include zinc therapy, indicating the European population, specifically children may not be zinc deficient like that of many developing nations such as Bangladesh [17]. Bangladesh also do not have a national guideline for child diarrhoeal management, similar to most developing countries. Most hospitals have developed their own protocol which are adopted from WHO guidelines on diarrhoeal management and therefore includes ORS for rehydration, zinc therapy and careful using antibiotics. Additionally, like most of its poor neighbors, Bangladesh do not have a robust health system and telephone consultation as exists in more developed nation, is an usual practice for general practitioners or even visit to qualified doctors is not always possible due to many access barriers especially in lowest rural tier [18]. Under such drawbacks, it is crucial to train and integrate paramedical workforce such as NGO workers in current health system to increase ORS and zinc therapy coverage in the country and provide effective management of diarrhoea to the rural population.

In Bangladesh, following the launch of the national zinc scale-up campaign in late 2006, repeat impact surveys were carried out in order to monitor diarrhoea management practices especially ORS and zinc usage in under-five children [19]. Before the initiation of the mass media campaign, caretakers' awareness of zinc as a treatment for childhood diarrhoea was under 5% in rural areas, however, increased to 55% by 12 months and 75% by 24 months [20]. On the other hand, zinc usage (any amount) as a treatment of childhood diarrhoea increased from under 5% to 13% during the same time period in rural areas.

Being a low income country, Bangladesh, since its independence in 1971, has been observed to have a pluralistic health system with a large segment of rural population still inclined to seek care from informal health sector providers such as traditional healers, pharmacists and ‘village doctors’ [21]–[23]. The fragmented health system also relies heavily on the several hundred or so local and national NGOs who have been playing a significant role in the health sector since the independence of the nation [24]. Under the latest national health, population and nutrition development program strategy of Bangladesh, which follows a sector-wide approach, the role of NGOs are recognized as more pivotal than ever, with increased Government-NGO collaboration [25]. Within the NGO sector, health services is directly provided in the community; however some NGOs also work closely with private sector unlicensed providers who are the preferred source of care seeking in rural communities in Bangladesh for childhood diarrhoea [26]. Involvements of NGOs working through networking with unlicensed providers are therefore a potentially important strategy to be included in the effort to bring zinc treatment of childhood diarrhoea to scale in Bangladesh.

The primary objective of the study was to determine whether a training intervention given to unlicensed health service providers by NGO providers' increases zinc coverage as an adjunct to ORS for the management of childhood diarrhoea in rural communities of Bangladesh.

## Materials and Methods

### Design and site

The protocol for this trial and supporting CONSORT checklist are available as supporting information; see Checklist S1 and Protocol S1. This uncontrolled, single-arm trial for a training and support intervention on diarrhoea outcomes was employed in a rural sub-district of Bangladesh during 2008. The study was conducted in Sreepur sub-district under Gazipur district of Bangladesh.

Two local NGOs with long working experience on providing health services in the chosen community were selected along with their catchment areas for the purpose of this study. The intervention area included 53 villages having a population of ∼95,000 against 49 villages in the control area with a population of ∼76,000. The curative health services in these sites were provided by unlicensed (local drug vendors, village practitioners, traditional healers), and licensed government, private or NGO health service providers. A cluster sampling design was employed and the required number of clusters (villages) was randomly selected from the total listed villages in both the intervention and control areas using a random number generating software/calculator. Baseline data was collected during February 2008 and endline data in August 2008.

The study evaluated the effect of intervention primarily on zinc coverage and secondarily on ORS and antibiotic use in childhood diarrhoea through community surveys. The study design was such that the participants (mothers/caretakers) were not aware of the intervention and hence was blinded. A baseline survey was conducted in the intervention and control villages concomitantly with the training of the health service providers in the intervention area and an endline survey around six months following the training. We have hypothesized that greater increased change in zinc coverage for diarrhoea management would be observed in the intervention when compared to control villages six months following the training.

#### Intervention

The intervention included orientation and training of the unlicensed health care providers (village doctors, drug vendors and traditional healers) regarding zinc treatment in childhood diarrhoea through NGO health care providers. In addition, the NGO conducted community-based promotion of zinc treatment in the intervention villages.

A package of training materials custom designed for the scale up of zinc for young children plus ORS was delivered using a training of the trainers'strategy. The package is described in Table 1.

**Table 1:** Training Package for health care providers in the intervention area.

**For licensed providers**
Refresher course and up-date on WHO/UNICEF guidelines for treatment of childhood diarrhea
Training videos (docu-drama)
Orientation/training materials (zinc babohar nirdeshika)
Frequently asked questions booklet
Follow-up support
**For unlicensed community health care providers**
Orientation/training materials (flip chart)
Training videos (docu-drama)
Zinc commercial (mass media campaign)
Refresher training and up-date on WHO/UNICEF guidelines for treatment of childhood diarrhea
Frequently asked questions booklet
Follow-up support

After the initial training to NGO service providers in the intervention areas, subsequent training was delivered by the NGOs done through quarterly meetings and follow-up sessions with village practitioners throughout the intervention areas. This work was conducted by Sub-Assistant Medical Officers (SACMOs). In intervention villages there are at least four SACMOs who have training experience with village practitioners.

### Ethical approval

The study was reviewed and approved by the Research Review and Ethical Review Committees of the International Centre for Diarrhoeal Diseases Research, Bangladesh (icddr,b). Informed written consent was obtained in a prescribed form from the caretakers of the enrolled children. The Trial Registration number for this protocol is NCT02143921. It is to be mentioned that due to procedural and institutional reasons, we were only able to register the trial after completing the study.

### Sample size estimation

To detect a minimum 50% increase in zinc coverage for childhood diarrhoea in the intervention area with a level of confidence of 0.95, a power of 0.9 and assuming a 1.5 design effect adjustment for clustering, it was estimated 580 cases of childhood diarrhoea per group would be required.

We have randomly selected 28 out of 49 villages from the control NGO's catchment area and 28 out of 53 villages in the intervention NGO's catchment area to meet the sample size requirement during the baseline survey, whereas; 26 and 29 villages respectively were again randomly selected during the endline survey.

#### Sampling

Baseline and endline data has been collected from all households from the selected villages in both control and intervention areas. Within each village, a central starting point was chosen and door-to-door survey technique of households was implemented for screening and recruiting children with a current or recent episode of diarrhoea (lasting for ≥2 days) within two weeks prior to the survey. Thus, information from all children with diarrhoea in the selected clusters was recorded. However, if more than one child in the household was eligible, one was randomly chosen. The respondents of the survey were the mothers or other caretakers of the identified children of the selected households.

### Statistics

Data were entered and verified using SPSS version 10.5 and converted to Stata version 11.5 for all analyses. Data were checked for missing values and recoded to generate new categorical variables using cut-off values. The analyses carried out were both stratified by time of the survey (baseline and endline) and location of residence (intervention and control) and without any stratification. To assess differences in categorical outcomes, chi-square statistical comparisons of proportions with 95% confidence intervals, were calculated. Of particular interest was the identification of disparities in the use of zinc, ORS and antibiotic by area. Age of child, gender, type of diarrhoea, duration of diarrhoea, age of mother, parental year of schooling, parental occupation, type of toilet facility and weekly expenditure on food (own production/purchase) as proxy indicator of financial status were included in the bivariate analysis in order to identify significant predictors of zinc and antibiotic usage. Variables that were significant (p<0.05) in bivariate analyses were included in the multiple logistic regression analyses after controlling for area and time in order to assess independent association of the variables with zinc and antibiotic usage. All the analyses were done by taking cluster effect into account using ‘***svy***’ command in Stata.

## Results

**Figure 1:**
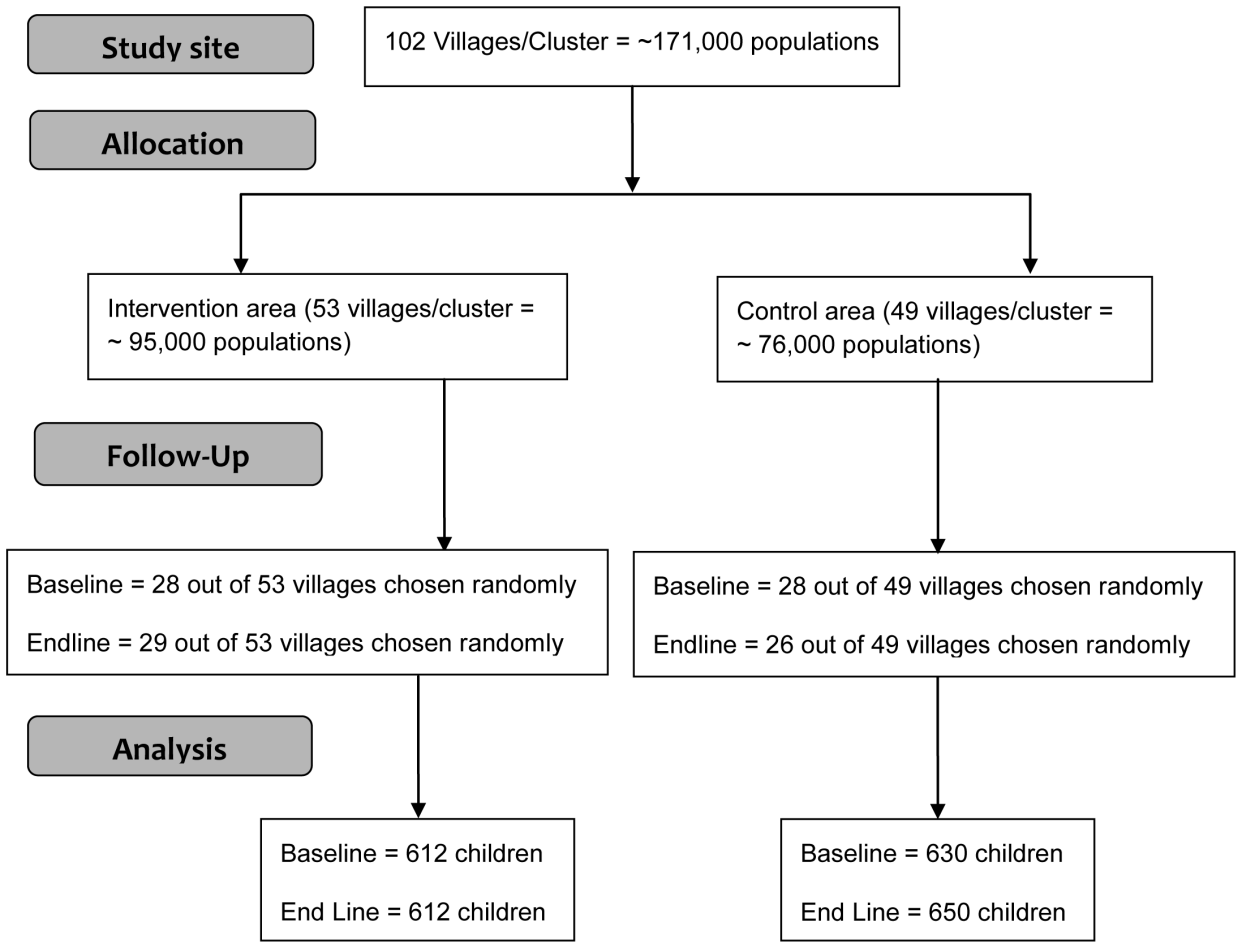
Trial Flowchart.

The survey result shows that at baseline, data of 630 and 612 children were collected from the control and intervention areas with current or recent episode of diarrhoea, whereas; at endline the figure was 650 and 612 children respectively. The trial flow chart is shown in Figure 1. Among the total cases (n = 2504), the most prevalent type of diarrhoea was ‘predominantly mucoid’ (63.7%) whereas 20.6% cases were ‘watery/loose’ and 15.7% cases had predominantly bloody diarrhoea. Among all cases, 71% children received ORS during diarrhoeal illness.

**Figure 2:**
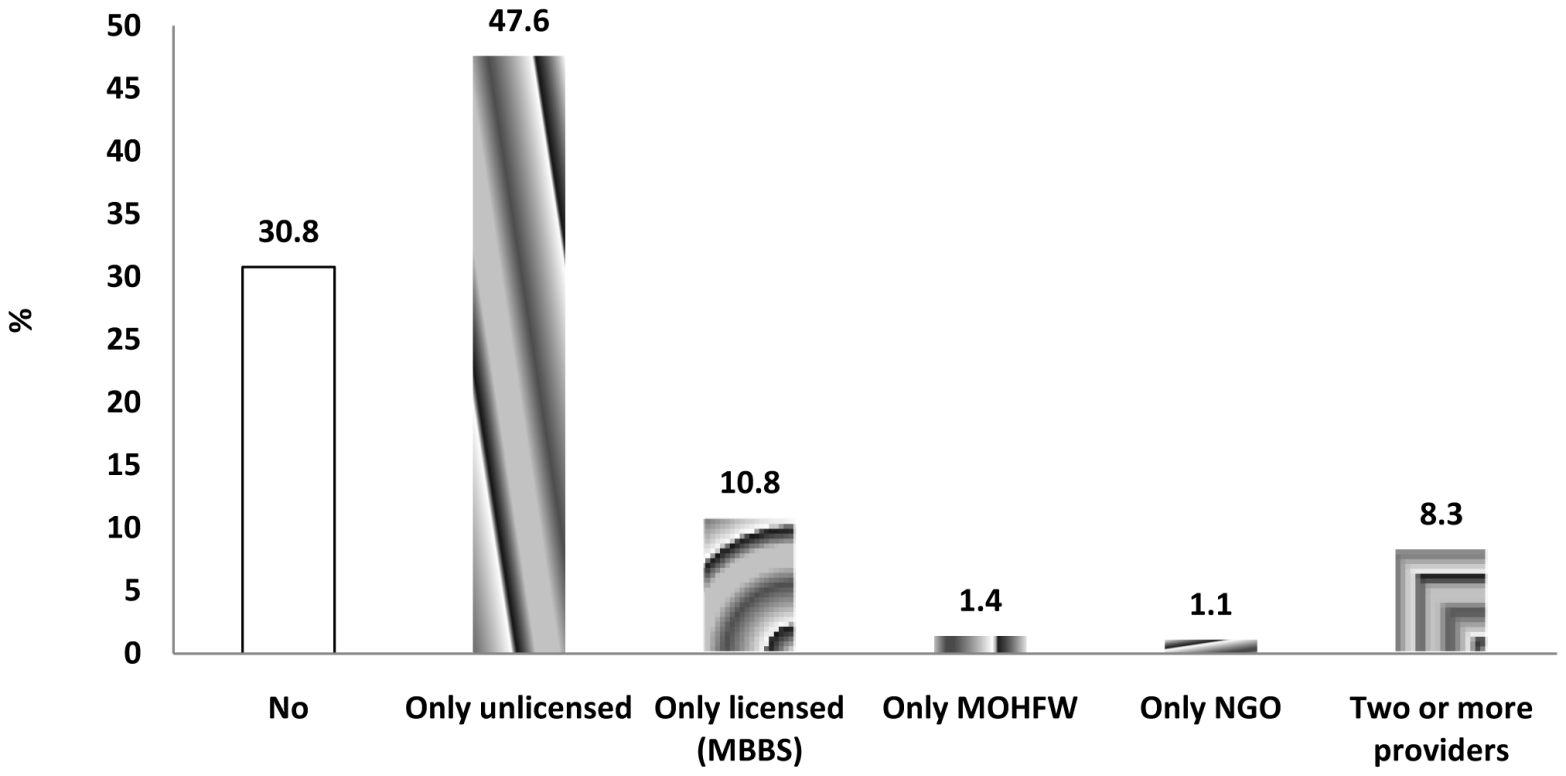
Health seeking behaviour during diarrhoeal illness among children 6–59 months by type of service providers.

Descriptive statistics of selected socio-demographic characteristics of households are presented in Table 2. No significant differences were found between control and intervention areas at baseline and endline in terms of age and sex of the study children, age of the mothers and type of toilet facility used by the household. Overall, irrespective of study area and time of the survey, 31% of the children did not seek treatment for diarrhoeal illness from any health service providers (Figure 2). Among the children who did seek treatment from service providers, most of them received treatment from unlicensed (e.g. village doctor, drug vendor, and traditional healers) private providers (68.7%). Almost 16% received treatment from licensed (MBBS) private providers whereas, only 1.6% and 2.1% went to NGO and government facilities respectively to receive treatment for their illness.

**Table 2:** Socio-demographic characteristics of the Control and Intervention areas.*

Variable	Baseline		Endline	
	Control Total n = 630	Intervention Total n = 612	Control Total n = 650	Intervention Total n = 612
	%	%	%	%
**Age of child in months**				
6–12	27.0	23.4	21.2	19.8
13–24	29.1	31.3	25.7	27.3
25–36	18.7	23.2	20.8	18.8
37–59	25.2	22.1	32.3	34.1
**Sex of child**				
Male	54	52.1	52.9	55.6
Female	46	47.9	47.1	44.4
**Age of mother in years**				
≤20	15.6	18.6	17.5	20.1
21–25	36.4	40.7	37.1	40.0
26–30	28.1	24.2	28.8	24.8
31–49	19.8	16.2	16.5	14.9
Missing	0.1	0.3	0.1	0.2
**Mother's year of schooling**				
No education	20.5	13.4	26.8	21.1
1–4 years	18.3	15.7	16.6	12.4
5–9 years	53.3	60.5	47.7	58.8
10–11 years	4.9	5.7	6.2	6.1
> = 12 years	2.9	4.4	2.6	1.3
Missing	0.1	0.3	0.1	0.3
**Father's year of schooling**				
No education	32.1	24.2	35.7	33.0
1–4 years	14.4	11.2	13.5	11.6
5–9 years	36.0	46.8	33.5	38.9
10–11 years	8.4	8.7	8.9	9.3
> = 12 years	7.3	8.9	8.0	6.5
Don't Know	1.8	0.2	0.3	0.7
**Type of toilet facility**				
No facility	3.8	5.7	2.8	2.3
Unimproved	65.9	59.2	62.0	59.0
Improved	30.3	35.1	35.2	38.7
**Weekly expenditure on food in BDT (Bangladesh Taka)**				
< = 500	6.8	4.4	2.0	2.6
501–1000	56.7	46.9	44.2	43.5
1001–1500	25.7	31.5	39.2	34.3
> = 1501	10.8	17.2	14.6	19.6

*Cluster adjusted analyses of all variables.

Table 3 presents the usage of ORS, zinc and antibiotic usage among children in the control and intervention areas. Compared to control area, ORS usage was significantly higher in the intervention area after 6 months of intervention (65.1 vs. 72.2%, p<0.01). Similarly, there was a significant difference in zinc usage between control and intervention areas (15.3% vs. 30.2%; p<0.01). After 6 months of intervention, the recommended usage of zinc for 10 days or more in under-5 diarrhoea was significantly higher among children in the intervention area compared to the control area (9.2% vs. 2.5%, p<0.01). Compared to the baseline prevalence, antibiotic usage increased more in the intervention area compared to control area at the endline.

**Table 3:** ORS, zinc and antibiotic usage in Control and Intervention areas.*

Variables	Baseline			Endline		
	Control % (95% CI)	Intervention % (95% CI)	*p*-value	Control % (95% CI)	Intervention % (95% CI)	*p*-value
**ORS usage**						
	**n = 630**	**n = 612**		**n = 650**	**n = 612**	
Yes	73.2 (69.1, 77.2)	74.4 (70.3, 78.3)	0.67	65.1 (61.2, 68.9)	72.2 (69, 75.4)	<0.01
**Zinc usage of any duration (Tablet or Syrup)**						
	**n = 594**	**n = 608**		**n = 636**	**n = 609**	
Yes	23.9 (19.2, 28.7)	22 (17.2, 26.9)	0.57	15.3 (13.1, 17.4)	30.2 (25.2, 35.3)	<0.01
**Duration of zinc usage (Tablet or Syrup)**						
	**n = 594**	**n = 608**		**n = 636**	**n = 609**	
No use	76.9 (72.2, 81.6)	78.5 (73.7, 83.3)	0.83	84.8 (82.6, 86.9)	69.8 (64.7, 74.8)	<0.01
1–3 day	8.8 (6.7, 10.9)	8.4 (5.7, 11.1)		6.6 (4.8, 8.5)	9.2 (7.1, 11.3)	
4–9 day	11.3 (8.4, 14.2)	9.7 (6.5, 12.9)		6.1 (4.3, 8)	11.8 (8.7, 15)	
≥10 day	3 (1.4, 4.7)	3.5 (1.7, 5.2)		2.5 (0.9, 4.2)	9.2 (6.9, 11.5)	
**Duration of zinc usage (Tablet)**						
	**n = 594**	**n = 608**		**n = 636**	**n = 609**	
No use	84.5 (80.2, 88.8)	83.9 (79.4, 88.3)	0.78	87.7 (85.6, 89.9)	76.4 (71.8, 80.9)	<0.01
1–3 day	5.7 (3.5, 7.9)	7.1 (4.2, 9.9)		6 (4.7, 7.3)	8.9 (6.7, 11)	
4–9 day	7.6 (4.7, 10.4)	6.6 (3.9, 9.2)		3.9 (2.5, 5.4)	6.9 (4.5, 9.3)	
≥10 day	2.2 (0.9, 3.5)	2.5 (1.2, 3.7)		2.4 (0.8, 3.9)	7.9 (5.4, 10.3)	
**Antibiotic usage**						
	**n = 623**	**n = 611**		**n = 639**	**n = 606**	
Yes	24.7 (20.5, 28.9)	23.6 (19.2, 27.9)	0.70	28.8 (26.2, 31.4)	36.3 (31.6, 41)	<0.01

*Cluster adjusted analyses of all variable.

Table 4 depicts the results of multiple logistic regression analyses exploring the relationship of potential factors with zinc usage. The older children aged 25–59 months were less likely to use zinc during diarrhoeal illness compared to younger children aged 6–12 months. On the other hand, children with a higher duration (4 to 10+ days) of diarrhoeal illness were more likely to use zinc compared to children with lower duration of illness (1–3 days). The other significant predictors of zinc usage were type of diarrhoea, parental year of schooling as well as ORS and antibiotic usage whereas, age of mother, mother occupation, sex of child, father occupation, type of toilet facility and weekly expenditure on food were insignificant to predict zinc usage.

**Table 4:** Predictors of zinc usage in childhood diarrhoea after controlling area and time variation.*

Variable#	OR (95% CI)	*p*-value
**Age of child in months**		
6–12	Reference	
13–24	1.13 (0.86, 1.48)	0.37
25–36	0.74 (0.55, 0.98)	0.04
37–60	0.39 (0.26, 0.59)	<0.01
**Type of diarrhoea**		
Watery/Loose	Reference	
Predominantly mucoid	0.0.6 (0.47, 0.78)	<0.01
Predominantly bloody	0.65 (0.48, 0.89)	<0.01
**Duration of diarrhoea**		
1–3 days	Reference	
4–9 days	1.89 (1.49, 2.4)	<0.01
> = 10 days	3.76 (2.41, 5.88)	<0.01
**Father's year of schooling**		
No education	Reference	
1–4 years	0.93 (0.66, 1.3)	0.67
5–9 years	1.07 (0.82, 1.4)	0.62
10–11 years	1.71 (1.16, 2.53)	<0.01
> = 12 years	1.03 (0.67, 1.58)	0.9
**Mother's year of schooling**		
No education	Reference	
1–4 years	1.11 (0.75, 1.66)	0.59
5–9 years	1.55 (1.1, 2.19)	0.01
10–11 years	2.1 (1.34, 3.29)	<0.01
> = 12 years	3.36 (1.8, 6.26	<0.01
**ORS usage**		
No	Reference	
Yes	5.83 (3.96, 8.61)	<0.01
**Antibiotic usage**		
No	Reference	
Yes	1.75 (1.35, 2.26)	<0.01

*All analyses were adjusted for cluster effect.

# Variables (age of child, type of diarrhoea, duration of diarrhoea, age of mother, Father's year of schooling, Mother's year of schooling, mother occupation, ORS usage and antibiotic usage) significant (p<0.05) in bivariate analysis (simple logistic regression) were used in multiple logistic regression analyses.

The results of multiple logistic regression analyses showing significant predictors of antibiotic usage are presented in Table 5. The children aged over 12 months were less likely to use antibiotic during diarrhoeal illness compared to younger children aged 6–12 months. However, children were more likely to use antibiotic who suffered predominately from bloody diarrhoea compared to watery/loose diarrhoea as well as with duration of diarrhoea for 4–9 days compared to 1–3 days. The other significant predictors of antibiotic usage were father's year of schooling, ORS usage and zinc usage.

**Table 5:** Predictors of antibiotic usage in childhood diarrhoea after controlling area and time variation.*

Variable#	OR (95% CI)	*p*-value
**Age of child in months**		
6–12	Reference	
13–24	0.66 (0.48, 0.91)	0.01
25–36	0.69 (0.51, 0.93)	0.02
37–60	0.55 (0.37, 0.84)	<0.01
**Type of diarrhoea**		
Watery/Loose	Reference	
Predominantly mucoid	1.02 (0.79, 1.3)	0.9
Predominantly bloody	1.79 (1.33, 2.43)	<0.01
**Duration of diarrhoea**		
1–3 days	Reference	
4–9 days	1.92 (1.58, 2.33)	<0.01
> = 10 days	2.78 (1.99, 3.87)	<0.01
**Father's year of schooling**		
No education	Reference	
1–4 years	1.04 (0.71, 1.51)	0.84
5–9 years	1.43 (1.07, 1.92)	0.02
10–11 years	1.83 (1.24, 2.72)	<0.01
> = 12 years	1.62 (1.07, 2.47)	0.02
**ORS usage**		
No	Reference	
Yes	2.07 (1.64, 2.63)	<0.01
**Zinc usage**		
No	Reference	
Yes	1.75 (1.36, 2.27)	<0.01

*All analyses were adjusted for cluster effect.

# Variables (age of child, type of diarrhoea, duration of diarrhoea, age of mother, father education, mother education, mother occupation, ORS usage and antibiotic usage) significant (p<0.05) in bivariate analysis (simple logistic regression) were included in multiple logistic regression analyses.

## Discussion

A simple training intervention to unlicensed private providers on diarrhoeal management by local NGOs had significantly increased zinc usage in a rural community compared to nonintervened control area. The study was conducted in 2008 after launching in late 2006 of the national campaign to scale up zinc treatment of childhood diarrhoea in Bangladesh. Two years after the inauguration of zinc program, the national prevalence of zinc usage in under-5 children was 9–13% in rural areas [19]. While in this study, we found that after 6 months of training intervention and community sensitization, zinc usage was increased to 30% in the intervention area from the baseline usage of 22% and in the non-intervention area it was reduced to 15% from the baseline usage of 24%. More importantly, the recommended 10 days zinc usage for diarrhoeal management was increased by 7% in the intervention area at the endline. As expected, we demonstrated a significant improvement in ORS usage following the NGO's training intervention to the unlicensed Health Care Providers.

Our finding clearly states the importance of training to the informal health care providers for overall zinc treatment and compliance to 10 days zinc treatment in addition to ORS for childhood diarrhoea management in the rural areas. Rural Bangladesh is facing a shortage of qualified physician (MBBS or above) over a long period of time and the shortage is less likely to be filled in near future [27]. Therefore, most people in the rural communities had to rely on privately owned local drug outlets where unlicensed health care providers such as drug sellers/vendors and village doctors are providing services. In 2006, Larson CP et al.; reported a dominance of private sector in providing health care delivery for diarrhoeal diseases both in urban and rural communities [26] while unlicensed health care providers remains the first point of privately delivering any health care services in most cases in the rural areas [27]. Training intervention to the unlicensed health care providers and community health workers for improving health is well acknowledged in many developing countries including Bangladesh [28]. Successful training and involvement of these health cadres are now a national policy for tuberculosis control in Bangladesh [29], [30].

It can be assumed that there is a difference in knowledge and awareness between unlicensed urban and rural health care providers as well as the urban and rural population. A previous study demonstrates a difference in awareness about zinc treatment for diarrhoeal diseases in children between urban and rural caregivers [20]. Since, the study area is very close to the capital city of the country therefore, it is more likely that the unlicensed health care providers in these areas are privileged for exchanging updated health related information including zinc treatment for diarrhoea in children. Also, the community dwellers are similarly privileged for receiving mass media and other sources of information compared to other rural, remote hard to reach areas. Despite, the above mentioned minor limitations, the findings can be generalized in context of Bangladesh. As the population in Bangladesh is rather homogenous in terms of culture and social characteristics, we do not expect too much variation in statistical findings if any other intervention areas are chosen. It is also to be notified that our study staff has confirmed no report of adverse effect occurred to any participants during the trial period due to the intervention.

We found 12.7% increment in antibiotic usage at endline in the intervention area vs. 4.1% increment in the control area compared to the baseline usage (23.6% in intervention vs. 24.7% in control area). The rate of antibiotic use was not much different in comparison to national usage of antibiotic for diarrhoeal diseases which was 31 and 36% in female and male children respectively in the rural areas [26]. In this study, the use of antibiotic was higher among younger children and those who were suffering from bloody diarrhoea. However, rational use of antibiotic is indicated in bloody diarrhoea while the reason for higher use of antibiotic in younger children is unclear.

The training intervention repeatedly emphasized that zinc treatment is an adjunct therapy to ORS for the management of childhood diarrhoea. Given this, we observed an increased utilization of ORS in the intervention area (72%) than control area (65%). As illustrated before that the intervention and control areas are rural settings though very close to the capital city and ORS usage in both these areas were comparable to the overall national ORS usage during concurrent time period (52–68%) in rural areas of Bangladesh [19].

Overall, the training intervention to the unlicensed health care providers is very effective in terms of zinc treatment coverage and compliance to 10 days zinc treatment, increasing ORS usage. This training intervention, can be seen as capacity building for non-professionals, who would be able to provide diarrhoeal management for established cases, and for more complicated cases referral would be done to nearest Government Health Care facility, and hence would provide sufficient pace, if scaled-up, to the nation's target to reduce childhood diarrhoeal mortality and morbidity and to achieve Millennium Development Goal (MDG) 4. Nevertheless, training intervention should be critically emphasized on the rational use of antibiotic in diarrhoea in young children.

## Conclusions

The study results shows that, a simple inexpensive training intervention for the unlicensed health care providers through NGO network increases the use of zinc for diarrhoea management. These efforts therefore need to continue with non-sector (Partnership of NGOs and Private Health Care Providers) provider for successful nationwide zinc scaling up.

### Learning

This study will contribute to effort of scaling up zinc by employing an existing network of NGO providers in the training of unlicensed providers.

## Supporting Information

Checklist S1 **CONSORT checklist of the clinical trial.**
(DOC)

Protocol S1 **Impact of an NGO training and support intervention on private sector provider diarrhea management practices.**
(DOC)

## References

[1] Walker CLF, Perin J, Aryee MJ, Boschi-Pinto C, Black RE (2012) Diarrhoea incidence in low-and middle-income countries in 1990 and 2010: a systematic review. BMC Public Health 12: 220. 22436130 10.1186/1471-2458-12-220 PMC3323412

[2] Bajait C, Thawani V (2011) Role of zinc in pediatric diarrhoea. Indian journal of pharmacology 43: 232. 21713083 10.4103/0253-7613.81495 PMC3113371

[3] Walker CLF, Black RE (2010) Zinc for the treatment of diarrhoea: effect on diarrhoea morbidity, mortality and incidence of future episodes. International Journal of Epidemiology 39: i63–i69. 20348128 10.1093/ije/dyq023 PMC2845862

[4] Wardlaw T, Salama P, Brocklehurst C, Chopra M, Mason E (2010) Diarrhoea: why children are still dying and what can be done. Lancet 375: 870–872. 19833382 10.1016/S0140-6736(09)61798-0

[5] Liu L, Johnson HL, Cousens S, Perin J, Scott S, et al (2012) Global, regional, and national causes of child mortality: an updated systematic analysis for 2010 with time trends since 2000. The Lancet 379: 2151–2161. 10.1016/S0140-6736(12)60560-1 22579125

[6] Boschi-Pinto C, Velebit L, Shibuya K (2008) Estimating child mortality due to diarrhoea in developing countries. Bulletin of the World Health Organization 86: 710–717. 18797647 10.2471/BLT.07.050054 PMC2649491

[7] Farthing M, Lindberg G, Dite P (2010) World gastroenterology organisation practice guideline: acute diarrhoea. 2008. Available: http://www.worldgastroenterology. org/assets/downloads/en/pdf/guidelines/01_acute_diarrhoea pdf. Accessed 29.

[8] Ahs JW, Tao W, Löfgren J, Forsberg BC (2010) Diarrhoeal Diseases in Low- and Middle-Income Countries: Incidence, Prevention and Management. The Open Infectious Diseases Journal 4: 113–124.

[9] WHO U (2004) WHO-UNICEF Joint statement on the clinical management of acute diarrhoea. World Health Assembly Geneva.

[10] Bhutta Z, Black R, Brown K, Gardner JM, Gore S, et al (1999) Prevention of diarrhoea and pneumonia by zinc supplementation in children in developing countries: pooled analysis of randomized controlled trials. The Journal of pediatrics 135: 689–697. 10586170 10.1016/s0022-3476(99)70086-7

[11] Bhutta ZA, Bird SM, Black RE, Brown KH, Gardner JM, et al (2000) Therapeutic effects of oral zinc in acute and persistent diarrhoea in children in developing countries: pooled analysis of randomized controlled trials. The American journal of clinical nutrition 72: 1516–1522. 11101480 10.1093/ajcn/72.6.1516

[12] Lazzerini M, Ronfani L (2008) Oral zinc for treating diarrhoea in children. Cochrane Database Syst Rev 3. 10.1002/14651858.CD005436.pub2 18646129

[13] Jones G, Steketee RW, Black RE, Bhutta ZA, Morris SS (2003) How many child deaths can we prevent this year? Lancet 362: 65–71. 12853204 10.1016/S0140-6736(03)13811-1

[14] Thapar N, Sanderson IR (2004) Diarrhoea in children: an interface between developing and developed countries. The Lancet 363: 641–653. 10.1016/S0140-6736(04)15599-2 14987892

[15] Szajewska H, Hoekstra JH, Sandhu B (2000) Management of acute gastroenteritis in Europe and the impact of the new recommendations: a multicenter study. Journal of Pediatric Gastroenterology and Nutrition 30: 522–527. 10817282 10.1097/00005176-200005000-00011

[16] Guarino A, Albano F, Ashkenazi S, Gendrel D, Hoekstra JH, et al (2008) European Society for Paediatric Gastroenterology, Hepatology, and Nutrition/European Society for Paediatric Infectious Diseases evidence-based guidelines for the management of acute gastroenteritis in children in Europe: executive summary. Journal of pediatric gastroenterology and nutrition 46: 619–621. 18493225 10.1097/MPG.0b013e31816e219e

[17] Ahmed T, Mahfuz M, Ireen S, Ahmed AS, Rahman S, et al (2012) Nutrition of children and women in Bangladesh: trends and directions for the future. Journal of health, population, and nutrition 30: 1. 10.3329/jhpn.v30i1.11268 PMC3312353 22524113

[18] Parkhurst JO, Rahman SA, Ssengooba F (2006) Overcoming access barriers for facility-based delivery in low-income settings: insights from Bangladesh and Uganda. Journal of health, population, and nutrition 24: 438. PMC3001147 17591340

[19] Larson CP, Saha UR, Nazrul H (2009) Impact monitoring of the national scale up of zinc treatment for childhood diarrhoea in Bangladesh: repeat ecologic surveys. PLoS medicine 6: e1000175. 19888335 10.1371/journal.pmed.1000175 PMC2765636

[20] Larson CP, Koehlmoos TP, Sack DA (2012) Scaling up zinc treatment of childhood diarrhoea in Bangladesh: theoretical and practical considerations guiding the SUZY Project. Health policy and planning 27: 102–114. 21343236 10.1093/heapol/czr015

[21] Ahmed SM (2005) Exploring health-seeking behaviour of disadvantaged populations in rural Bangladesh: Institutionen för folkhälsovetenskap/Department of Public Health Sciences.

[22] Ahmed SM, Evans TG, Standing H, Mahmud S (2013) Harnessing pluralism for better health in Bangladesh. The Lancet 382: 1746–1755. 10.1016/S0140-6736(13)62147-9 24268003

[23] Ahmed SM, Hossain MA, Chowdhury MR (2009) Informal sector providers in Bangladesh: how equipped are they to provide rational health care? Health Policy and Planning 24: 467–478. 19720721 10.1093/heapol/czp037

[24] Mercer A, Khan MH, Daulatuzzaman M, Reid J (2004) Effectiveness of an NGO primary health care programme in rural Bangladesh: evidence from the management information system. Health Policy and Planning 19: 187–198. 15208275 10.1093/heapol/czh024

[25] Ministry of Health and Family Welfare B (2011) Health, Population and Nutrition Sector Development Program (2011–2016) Program Implementation Plan. In: Welfare PWMoHaF, editor: Government of the People's Republic of Bangladesh.

[26] Larson CP, Saha UR, Islam R, Roy N (2006) Childhood diarrhoea management practices in Bangladesh: private sector dominance and continued inequities in care. International Journal of Epidemiology 35: 1430–1439. 16997849 10.1093/ije/dyl167

[27] Mahmood SS, Iqbal M, Hanifi S, Wahed T, Bhuiya A (2010) Are ‘Village Doctors’ in Bangladesh a curse or a blessing? BMC international health and human rights 10: 18. 20602805 10.1186/1472-698X-10-18 PMC2910021

[28] Oshiname FO, Brieger WR (1992) Primary care training for patent medicine vendors in rural Nigeria. Social science & medicine 35: 1477–1484. 1485195 10.1016/0277-9536(92)90050-z

[29] Chowdhury AMR, Chowdhury S, Islam MN, Islam A, Vaughan JP (1997) Control of tuberculosis by community health workers in Bangladesh. The Lancet 350: 169–172. 10.1016/S0140-6736(96)11311-8 9250184

[30] Salim H, Uplekar M, Daru P, Aung M, Declercq E, et al (2006) Turning liabilities into resources: informal village doctors and tuberculosis control in Bangladesh. Bulletin of the World Health Organization 84: 479–484. 16799732 10.2471/blt.05.023929 PMC2627374

