# Supplementary material for: Impact of NGO Training and Support Intervention on Diarrhoea Management Practices in a Rural Community of Bangladesh: An Uncontrolled, Single-Arm Trial
Source: PLoS One. 2014 Nov 14;9(11):e112308. doi: 10.1371/journal.pone.0112308 (PMC4232353; doi:10.1371/journal.pone.0112308)
Supplement: Protocol S1 — Impact of an NGO training and support intervention on private sector provider diarrhea management practices. (DOC) [file pone.0112308.s002.doc]

| 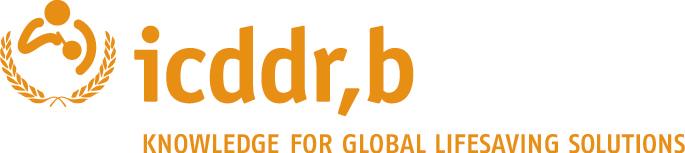 | | | | RRC APPLICATION FORM | | |
| --- | --- | --- | --- | --- | --- | --- |
| RESEARCH PROTOCOL **NUMBER: 2007-051** | **FOR OFFICE USE ONLY** | | | | | |
|  | RRC Approval: | | | Yes / | No | Date:10/24/2007 |
|  | ERC Approval: | | | Yes / | No | Date:12/30/2007 |
|  | AEEC Approval: | | | Yes / | No | Date: |
| **Protocol Title:** Impact of an NGO training and support intervention on private sector provider diarrhea management practices | | | | | | |
| **Short title (in 50 characters including space):** Impact of training on diarrhoea management | | | | | | |
| **Theme: (Check all that apply)**  X Nutrition  Emerging and Re-emerging Infectious Diseases  Population Dynamics  Reproductive Health  Vaccine Evaluation  HIV/AIDS | | | Environmental Health  X Health Services  X Child Health  Clinical Case Management  Social and Behavioural Sciences | | | |
| **Key words:** zinc, scaling up, training, diarrhoea, management, service providers. | | | | | | |
| **Relevance of the Protocol:**  Alternative, sustainable interventions in support of zinc treatment for childhood diarrhea need to be identified. Within the NGO sector, direct provision of health services is provided, however some NGOs also work closely with the private sector providers. Hypothetically, this avoids unnecessary duplication of effort and enhances the quality of the care provided in the private sector. This study will provide objective evidence of the impact of this approach, using the scale-up of zinc for the treatment of childhood diarrhoea as a test case. The study will be assessing NGO impact among unregulated (unlicensed) rural service providers. Further, this study will serve to assess the value of this model of scaling up activities in the non-state sector with a particular eye toward more generalizable and replicable improvement in quality of care strategies between NGOs and unlicensed private providers. | | | | | | |
| **Centre’s Priority** **(as per Strategic Plan, to be imported from the attached Separate Word Sheet):**  To evaluate alternative service strategies and provide evidence to better define health services. | | | | | | |
| **Programmes:**  X Child Health Programme  X Nutrition Programme  Programme on Infectious Diseases & Vaccine Science  Poverty and Health Programme | | | X Health and Family Planning Systems Programme    Population Programme  Reproductive Health Programme  HIV/AIDS Programme | | | |
| **Principal Investigator (Should be a Centre’s staff)**  **Charles P. Larson**  **Address (including e-mail address):**  ICDDR,B, email: clarson@icddrb.org | | | **DIVISION:**  CSD  LSD  X HSID  PHSD | | | |
| **Co-Principal Investigator(s): Internal**  Tracey Koehlmoos | | | | | | |
| **Co-Principal Investigator(s): External:**  (Please provide full official address including e-mail address and Gender) | | | | | | |
| **Co-Investigator(s): Internal:**  A. Shafiqur Rahman | | | | | | |
| **Co-Investigator(s): External**  (Please provide full official address including e-mail address and Gender) | | | | | | |
| **Student Investigator(s): Internal (Centre’s staff):** | | | | | | |
| **Student Investigator(s): External:**  (Please provide full address of educational institution and Gender) | | | | | | |
| **Collaborating Institute(s):** Please Provide full address   | Country | Bangladesh | | --- | --- | | Contact person | Dr. Selina Amin | | Department  (including Division, Centre, Unit) | Health Advisor | | Institution  (with official address) | Plan Bangladesh, House CWN (B) Rd. 35, Gulshan-2, Dhaka 1212 | | Directorate  (in case of GoB i.e. DGHS) |  | | Ministry (in case of GoB) |  |   **Institution # 1** | | | | | | |
| **Institution # 2**   | Country | Bangladesh | | --- | --- | | Contact person | Dr. Dibalok Singha | | Department  (including Division, Centre, Unit) | Executive Director | | Institution  (with official address) | Dushtho Sasthya Kendra (DSK), House # 741, Road # 9, Baitul Aman Housing Society, Adabar, Dhaka 1207 | | Directorate  (in case of GoB i.e. DGHS) |  | | Ministry (in case of GoB) |  | | | | | | | |
| **Institution # 3**   | Country | Bangladesh | | --- | --- | | Contact person | Dr. Mohibullah Khondokar | | Department  (including Division, Centre, Unit) | PD | | Institution  (with official address) | Gonoshasthya Kendra, Tengra, Sreepur, Gazipur | | Directorate  (in case of GoB i.e. DGHS) |  | | Ministry (in case of GoB) |  |   Note: If more than 3 collaborating institutions are involved in the research protocol, additional block(s) can be inserted to mention its/there particular(s). | | | | | | |
| **Population: Inclusion of special groups (Check all that apply):**   | Gender  X Male  X Female  Age  X 0 – 4 years  5 – 9 years  10 – 19 years  X 20 – 64 years  65 + | Pregnant Women  Fetuses  Prisoners  Destitutes  Service Providers  Cognitively Impaired  CSW  Others (specify      )  Animal | | --- | --- |   NOTE It is the policy of the Centre to include men, women, and children in all research projects involving human subjects unless a clear and compelling rationale and justification (e.g. gender specific or inappropriate with respect to the purpose of the research) is there. Justification should be provided in the `Sample Size’ section of the protocol in case inclusiveness of study participants is not proposed in the study. | | | | | | |
| **Project/study Site (Check all the apply):**  Dhaka Hospital  Matlab Hospital  Matlab DSS Area  Matlab non-DSS Area  Mirzapur  Dhaka Community  Chakaria  Abhoynagar | | Mirsarai  Patyia  X Other areas in Bangladesh  Outside Bangladesh  Name of Country:  Multi Centre Trial  (Name other countries involved): | | | | |
| Type of Study (Check all that apply):   | Case Control Study  X Community-based Trial/Intervention  Program Project (Umbrella)  Secondary Data Analysis  Clinical Trial (Hospital/Clinic)  Family Follow-up Study | Cross Sectional Survey  Longitudinal Study (cohort or follow-up)  Record Review  Prophylactic Trial  Surveillance/Monitoring  X Others: Controlled before-after study | | --- | --- |   NOTE: Does the study meet the definition of clinical studies/trials given by the International Committee of Medical Journal Editors (ICMJE)? Yes  No    Please note that the ICMJE defined clinical trial as “*Any research project that prospectively assigns human subjects to intervention and comparison groups to study the cause-and-effect relationship between a medical intervention and a health outcome*”.    If YES, after approval of the ERC, the PI should complete and send the relevant form to provide required information about the research protocol to the Committee Coordination Secretariat for registration of the study into websites, preferably at the [www.clinicaltrials.gov](http://www.clinicaltrials.gov/). It may please be noted that the PI would require to provide subsequent updates of the research protocol for updating protocol information in the website. | | | | | | |
| **Targeted Population (Check all that apply):**  No ethnic selection (Bangladeshi)  Bangalee  Tribal group | | Expatriates  Immigrants  Refugee | | | | |
| **Consent Process (Check all that apply):**  Written  Oral  None | | X Bengali Language  English Language | | | | |
| **Proposed Sample Size:** 1160 in baseline and end line will be interviewed  Sub-group (Name of subgroup (e.g. Men, Women) and Number   | Name | Number | Name | Number | | --- | --- | --- | --- | | (1) households | 1160 | (3) NGO providers | 8 | | (2) private sector providers | 20 |  |  |   Total sample size: 1188 | | | | | | |
| **Determination of Risk: Does the Research Involve (**Check all that apply**):**   | Human exposure to radioactive agents?  Fetal tissue or abortus?  Investigational new device?  (specify:      )  Existing data available from Co-investigator | Human exposure to infectious agents?  Investigational new drug  Existing data available via public archives/sources  Pathological or diagnostic clinical specimen only  Observation of public behaviour  New treatment regime | | --- | --- | | | | | | | |
| **Could the information recorded about the individual if it became known outside of the research:**   | Yes  X | No | Is the information recorded in such a manner that study participants can be identified from information provided directly or through identifiers linked to the study participants? | | --- | --- | --- | | Yes | No  X | Does the research deal with sensitive aspects of the study participants’ behaviour; sexual behaviour, alcohol use or illegal conduct such as drug use? |  | Yes | No  X | Place the study participants at risk of criminal or civil liability? | | --- | --- | --- | | Yes | No  X | Damage the study participants’ financial standing, reputation or employability, social rejection, lead to stigma, divorce etc.? | | | | | | | |
| **Do you consider this research (Check one):**  Greater than minimal risk X No more than minimal risk  Only part of the diagnostic test  Minimal Risk is "a risk where the probability and magnitude of harm or discomfort anticipated in the proposed research are not greater in and of themselves than those ordinarily encountered in daily life or during the performance of routine physical, psychological examinations or tests. For example, risk of drawing a small amount of blood from a healthy individual for research purposes is no greater than the risk of doing so as a part of routine physical examination". | | | | | | |
| **Yes/ No**  X  Is the proposal funded?  If yes, sponsor Name: (1) Bill & Melinda Gates Foundation    (2) | | | | | | |
| **Yes/No**  X Is the proposal being submitted for funding?  If yes, name of funding agency: (1)  (2) | | | | | | |
| Do any of the participating investigators and/or member(s) of their immediate families have an equity relationship (e.g. stockholder) with the sponsor of the project or manufacturer and/or owner of the test product or device to be studied or serve as a consultant to any of the above?  ***IF YES, a written statement of disclosure to be submitted to the Centre’s Executive Director.***  Dates of Proposed Period of Support Cost Required for the Budget Period ($)  (Day, Month, Year - DD/MM/YY)   | **Years** | **Direct Cost** | **Indirect Cost** | **Total Cost** | | --- | --- | --- | --- | | **Year-1** | 71,674 | - | 71,674 | | **Total** | 71,674 | - | 71,674 |     Beginning Date : November 1, 2007    End Date : May 31, 2008 | | | | | | |
| **Certification by the Principal Investigator**  I certify that the statements herein are true, complete and accurate to the best of my knowledge. I am aware that any false, fictitious, or fraudulent statements or claims may subject me to criminal, civil, or administrative penalties. I agree to accept the responsibility for the scientific conduct of the project and to provide the required progress reports including updating protocol information in the SUCHONA (Form # 2) if a grant is awarded as a result of this application.  ___________ ____________  **Signature of PI Date** | | | | | | |
| **Approval of the Project by the Division Director of the Applicant**  The above-mentioned project has been discussed and reviewed at the Division level as well by the external reviewers. The protocol has been revised according to the reviewers’ comments and is approved.   |  |  |  | | --- | --- | --- | | Name of the Division Director | Signature | Date of Approval | | | | | | | |

Table of Contents

[RRC APPLICATION FORM 1](#__RefHeading___Toc178476229)

[Project Summary 8](#__RefHeading___Toc178476230)

[Description of the Research Project 9](#__RefHeading___Toc178476231)

[Hypothesis to be Tested: 9](#__RefHeading___Toc178476232)

[Specific Aims: 9](#__RefHeading___Toc178476233)

[Specific Research objectives 9](#__RefHeading___Toc178476235)

[Background of the Project including Preliminary Observations 9](#__RefHeading___Toc178476236)

Research Design and Methods ..………………………………………………………………...12

[Sample Size Calculation and Outcome Variable(s) 14](#__RefHeading___Toc178476242)

[Sample size estimation for Survey 14](#__RefHeading___Toc178476243)

[Facilities Available 16](#__RefHeading___Toc178476249)

[Data Safety Monitoring Plan (DSMP) 16](#__RefHeading___Toc178476250)

[Data Analysis 16](#__RefHeading___Toc178476251)

[Ethical Assurance for Protection of Human Rights 17](#__RefHeading___Toc178476252)

[Use of Animals 17](#__RefHeading___Toc178476253)

[Literature Cited 18](#__RefHeading___Toc178476254)

[Dissemination and Use of Findings 20](#__RefHeading___Toc178476255)

[Collaborative Arrangements 20](#__RefHeading___Toc178476256)

[Biography of the Investigators 21](#__RefHeading___Toc178476257)

Budget Justification ..........................................................................................................................29

Other support.....................................................................................................................................31

[Appendix 1: Voluntary Consent Form 26](#__RefHeading___Toc178476258)

Appendix 2: Comments of External Reviewers............................................................................33

Appendix 3: Data collection tools.................................................................................................35

[Check-List......................................................................................................................................32](#__RefHeading___Toc178476264)

Check here if appendix is included

| Project Summary Describe in concise terms, the hypothesis, objectives, and the relevant background of the project. Also describe concisely the experimental design and research methods for achieving the objectives. This description will serve as a succinct and precise and accurate description of the proposed research is required. This summary must be understandable and interpretable when removed from the main application. |
| --- |
| Principal Investigator(s): Charles P. Larson |
| Research Protocol Title: Impact of an NGO training and support intervention on private sector provider diarrhea management practices |
| Total Budget US$: 71,674 Beginning Date : November 1, 2007 Ending Date: May 31, 2008 |
| Alternative, sustainable interventions in support of zinc treatment for childhood diarrhea need to be identified. Within the NGO sector, direct provision of health services is provided, however some NGOs also work closely with the private sector providers. Hypothetically, this avoids unnecessary duplication of effort and enhances the quality of the care provided in the private sector. This study will provide objective evidence of the impact of this approach, using the scale-up of zinc for the treatment of childhood diarrhoea as a test case. The study will be assessing NGO impact among unregulated (unlicensed), rural service providers. Further, this study will serve to assess the value of this model of scaling up activities in the non-state sector with a particular eye toward more generalizable and replicable improvement in quality of care strategies between NGOs and unlicensed private providers. This will be controlled before-after (CBA) study. An equivalent control area will be included in the study. The objective is to document changes in zinc and antibiotic as well as anti-diarrhoeal usage in childhood diarrhea following a scale-up intervention package delivered by a local NGO to local private providers. This will include sensitization, training and follow-up support. Impact will be assessed in terms of changes in zinc coverage and antibiotic use among households in the catchment population served by these providers. The study will be conducted in four unions located in Sreepur Upazila within Gazipur district, located north to Dhaka. Two of the unions, namely Rajabari and Prohladpur comprising 55 villages with 15,530 households and an estimated 76,150 population will serve as intervention area. The other two unions Telihat and Barmi will be taken as control sites. They contain 53 villages with 19,898 households and an estimated 99,000 population. The curative health services in these sites are provided by local drug vendors, village practitioners, traditional healers, and licensed government, private or NGO health service providers. Data will be collected through survey and in-depth interviews. Data will be entered and analyzed using SPSS version 12.0. Absolute counts, proportions, and means with 95% confidence interval will be calculated. These and regression analysis will be done using STATA version 9, cluster survey program, that accounts for potential within cluster homogeneity. Based upon the household surveys, the outcomes that will be calculated are: coverage-the proportion receiving zinc, ORS (zinc and ORS), and antibiotics or antidiarrhoeals and changes in coverage 6 months following the introduction of the intervention package, equity: who is receiving zinc by age, gender and socioeconomic status. For differences in categorical outcomes crude relative risks and 95% confidence intervals will be determined. It is expected that the study will help in increasing zinc coverage for the treatment of children under five years of age and decrease in the use of antibiotics and antidiarrhoeals within the NGO catchment population. |
| KEY PERSONNEL (List names of all investigators including PI and their respective specialties)     | **Name** | **Professional Discipline/ Specialty** | **Role in the Project** | | --- | --- | --- | | 1. Charles Larson | Division Director | Principal Investigator | | 2. Tracey Koehlmoos | Health Systems Researcher | Co-Principal Investigator | | 3. A. Shafiqur Rahman | Sr. Operations Researcher | Investigator | |

# Description of the Research Project

## Hypothesis to be Tested:

Concisely list in order, the hypothesis to be tested and the Specific Aims of the proposed study. Provide the scientific basis of the hypothesis, critically examining the observations leading to the formulation of the hypothesis.

That an NGO initiated sensitization, training and support package to scale up the prescribing of

zinc among NGO and private sector providers for the treatment of acute childhood diarrhea will,

within 4 months, result in:

H1. A significant increase in zinc coverage for the treatment of children under five years of age

within the NGO catchment population, and

H2. A significant decrease in the use of antibiotics and antidiarrhoeals

## Specific Aims:

Describe the specific aims of the proposed study. State the specific parameters, biological functions/ rates/ processes that will be assessed by specific methods.

# General objectives

To assess the impact of an NGO zinc scale up package developed to maximize compliance with WHO/UNICEF and MOHFW zinc treatment recommendations for acute childhood diarrhea (ACD)

# Specific Research objectives

Following the implementation of the proposed scale-up intervention package by a local NGO, Dushtha Shasthya Kendra (DSK).

- - to document changes in zinc coverage for ACD
  - to document changes in inappropriate use of antibiotics and antidiarrheals
  - to identify barriers/disincentives to the prescribing of zinc treatment among private providers.

## Background of the Project including Preliminary Observations

Describe the relevant background of the proposed study. Discuss the previous related works on the subject by citing specific references. Describe logically how the present hypothesis is supported by the relevant background observations including any preliminary results that may be available. Critically analyze available knowledge in the field of the proposed study and discuss the questions and gaps in the knowledge that need to be fulfilled to achieve the proposed goals. Provide scientific validity of the hypothesis on the basis of background information. If there is no sufficient information on the subject, indicate the need to develop new knowledge. Also include the significance and rationale of the proposed work by specifically discussing how these accomplishments will bring benefit to human health in relation to biomedical, social, and environmental perspectives.

Diarrhoeal disease is the second leading cause of under-five mortality in developing countries, accounting for 1.5 to 2 million deaths per year (1,2). The large majority of these deaths can be prevented by the timely use of oral rehydration solutions (ORS), zinc treatment and continued feeding practices (3).

## Zinc as a treatment for childhood diarrhoea:

Several randomized hospital and community-based trials have consistently demonstrated the efficacy of zinc treatment for acute or persistent diarrhoea in children under-five years of age (4,5,6,7,8,9).

Pooled or meta-analyses of published studies demonstrate that zinc supplementation reduces the duration and severity of acute diarrhoea and reduces the likelihood of a prolonged episode (10,11). It is estimated that the incidence and prevalence of childhood diarrhoea can be reduced by 18% (95% CI, 7-28%) and 28% (95% CI, 12-37%) respectively in zinc treated children (10). A community-based, effectiveness trial of zinc treatment for acute childhood diarrhea was carried out in the ICDDR,B rural field site located in Matlab. In this trial, in which children received daily zinc treatments with each episode of diarrhoea, the children in the zinc intervention group had a shorter duration of illness, a reduced likelihood of a repeat diarrhoea episode and reduced non-injury mortality. The reduction of mortality was substantial, being 50% (12).

WHO has estimated the global annual mortality burden attributable to zinc deficiency to be 750,000 deaths per year (13). It is anticipated that over one-half of these deaths could be averted through the successful application of zinc as a treatment for childhood diarrhea (14).

**WHO/UNICEF joint statement on diarrhoea management:**

Given this potential mortality reduction and the strength of the evidence at hand in support of zinc treatment, WHO/UNICEF issued a joint statement on updated guidelines for the management of childhood diarrhea in May, 2004(15). This includes the recommendation that all children under five be treated with zinc (20 mg/day if 6-59 months and 10 mg/day if less than 6 months of age) for 10 to 14 days. This recommendation is now a policy of the Ministry of Health and Family Welfare, with the slight modification to include children starting at two months of age.

The WHO/UNICEF joint statement calls for countries to assess progress in controlling diarrhoeal diseases, prioritize the availability of ORS and zinc supplements, strategize educating health workers at all levels about the use of zinc, and promote the availability of zinc further research into scaling up the use of zinc.

# Scaling up of Zinc:

Zinc scaling up efforts in Bangladesh focus on collaboration among ICDDR,B, the Ministry of Health and Family Welfare, ACME Laboratories and many other public and private sector organizations. The aim of this collaboration is to provide zinc treatment for diarrhoea on a national scale, targeting the entire under-five year-old population of Bangladesh. It has been estimated that zinc treatment could save the lives of up to 50,000 children per year in Bangladesh alone (12).

At present, the SUZY project has adopted the following scale-up strategies:

1. A mass media campaign
2. Provider promotion through ACME sales representatives
3. Orientation sessions with licensed providers
4. Training workshops for village doctors
5. Integration of zinc treatment into IMCI protocols (public sector)

The primary emphasis has been on reaching private providers through existing private sector channels. Thus far, we have not worked closely with the NGO sector. This could be an important,

additional strategy to further increasing zinc coverage across Bangladesh, in particular in communities highly reliant upon NGO services.

**Reduction of antibiotic usage for diarrhea:**

Antimicrobial and antidiarrhoeal drugs are unnecessary and potentially hazardous in the treatment of childhood diarrhoea. Numerous studies conducted around the globe have demonstrated that the use of zinc combined with ORS can reduce the inappropriate use of antibiotics. Baqui and colleagues found a statistically significant reduction in antibiotic use and the attitude for the treatment of childhood diarrhoea when zinc was introduced during a controlled, community-based study in Bangladesh (16). In India Bhandari et al. found a 34% reduction in the inappropriate use of tablets and a 64% reduction in the use of injections for the treatment of diarrhea after zinc was introduced (17).

**Rationale:**

In order to reach all children with diarrhoea, zinc treatment will need to be introduced and sustained within public, NGO and private service delivery systems. To improve zinc coverage, the Ministry of Health and Family Welfare (MOHFW) has initiated a program within the public sector that aims to distribute zinc, free of charge as a treatment of childhood diarrhea. However, a large population of the country, mostly the poor are served by NGO health services. Plan International, Bangladesh has been working for many years with children and families in some of the poorest communities, with aim of improving the quality of their lives, including their health. Across Bangladesh Plan works with 30 partner NGOs who provide community based services. These NGOs are highly respected within the communities they work, both among the general population and health providers. Whether these NGOs can significantly impact the diarrhoea treatment practices of private providers is unclear and should not be assumed. Past work carried out by the SUZY team with NSDP service providers plus local community sensitization and zinc promotion was not successful in improving zinc treatment coverage. The Plan model of having NGOs work with local providers is an alternative that deserves attention and validation of impact.

In Bangladesh, health seeking for childhood diarrhoea is dominated by utilization of private sector providers (18). Unlicensed providers (village doctors, drug sellers, and homeopaths) comprise more than 90% of the preferred source of care when help is sought.

The proposed study will be carried out in a rural community, where Dushtho Sasthya Kendra (DSK) [intervention area] and Ganosasthyo Kendra (GK) [control area], two partner NGOs of Plan International provide primary health care services along with other programs in Gazipur district, located north of Dhaka.

The proposed study will assess the impact of a zinc scale up intervention package that entails strong and sustained NGO-private sector collaboration. The aim is to build upon the existing mass media and provider promotion campaigns already implemented in Bangladesh, in the hope that this additional collaboration will maximize zinc treatment coverage for acute childhood diarrhoea (ACD). The impact of the zinc scale up package on inappropriate prescription/sale of antibiotics will also be assessed. The proposed study will assess the impact of a zinc scale up intervention package that entails strong and sustained NGO-private sector collaboration. We will determine whether the effort and expense of scaling up specifically with NGOs provides value added to the nation-wide programs described above as well as other changes to the public health system.

**Contribution to the Literature**

This impact study will contribute to the body of zinc/ORS and scaling up literature by employing an existing network of NGO providers in the training of unlicensed providers, not only on the use of zinc and ORS but also on the overall appropriate management of childhood diarrhea according to WHO/UNICEF standards.

**Research Design and Methods**

Describe in detail the methods and procedures that will be used to accomplish the objectives and specific aims of the project. Discuss the alternative methods that are available and justify the use of the method proposed in the study. Justify the scientific validity of the methodological approach (biomedical, social, or environmental) as an investigation tool to achieve the specific aims. Discuss the limitations and difficulties of the proposed procedures and sufficiently justify the use of them. Discuss the ethical issues related to biomedical and social research for employing special procedures, such as invasive procedures in sick children, use of isotopes or any other hazardous materials, or social questionnaires relating to individual privacy. Point out safety procedures to be observed for protection of individuals during any situations or materials that may be injurious to human health. The methodology section should be sufficiently descriptive to allow the reviewers to make valid and unambiguous assessment of the project.

**Study Design:** This will be controlled before-after (CBA) study. An equivalent control area will be included in the study. The objective is to document changes in zinc and antibiotic as well as anti-diarrhoeal usage in childhood diarrhoea following a scale-up intervention package delivered by a local NGO to local private providers. This will include sensitization, training and follow-up support. Impact will be assessed in terms of changes in zinc coverage and antibiotic use among households in the catchment population served by these providers.

## Study sites: The study will be conducted in four unions located in Sreepur Upazila within Gazipur district, located north to Dhaka. Two of the unions, namely Rajabari and Prohladpur comprising 55 villages with 15,530 households and an estimated 76,150 population will serve as intervention area. The other two unions Telihat and Barmi will be taken as control sites. They contain 53 villages with 19,898 households and an estimated 99,000 population. Local drug vendors, village practitioners, traditional healers, and licensed government, private or NGO health service providers provide the curative health services in these sites.

## Study populations

1. Care takers of the children aged 6 months to 5 years with a prevalent case of diarrhea at the

time of the household surveys (within the past 2 weeks). Within each site a systematic household

survey will be completed for the identification of any child 6 months to 5 years who has a

prevalent case of at least 2 days duration.

2. Health Care Providers

1. NGO licensed “sub-assistant community medical officers (SACMOs)
2. Private sector, unlicensed providers: village practitioners and traditional healers

There are eight SACMOs and one medical officer posted at static and satellite clinics per two unions. Within each site a census will be carried out to enumerate all private sector providers by type. 10 of each type will be randomly selected to complete in-depth interviews.

## Intervention Package:

- Training/orientation on diarrhea case management
- Follow-up health provider support

ICDDR,B will first train the trainers, i.e. the NGO health care providers who will implement the intervention package. These individuals will then conduct the training and support of other NGO staff and private unlicensed providers.

[note: we would have liked to include licensed providers, but the #s are insufficient]

The training/orientation materials to be used are summarized in the box below:

###### Orientation package/training program

For DSK Health care providers (MBBS & SACMO): provided by ICDDR,B study staff

- Refresher course and up-date on WHO/UNICEF guidelines for treatment of childhood diarrhoea
- Training videos (docu-drama)
- Orientation/training materials (zinc babohar nirdeshika)
- Frequently asked questions booklet
- Follow-up support

For 1^st^ line community health care providers (Village practitioner, drug venders, traditional healers): provided by DSK SACMO trainers

- Orientation/training materials (flip chart, zinc babohar nirdeshika)
- Training videos (docu-drama)
- Zinc commercial (mass media campaign)
- Refresher training and up-date on WHO/UNICEF guidelines for treatment of childhood diarrhea
- Frequently asked questions booklet
- Follow-up support

## The training provided by the NGOs will be done through quarterly meetings and follow-up sessions with village practitioners throughout the unions. This work will be done by Sub-Assistant Medical Officers (SACMOs). The SACMOs are graduates of diploma courses at government medical schools. This course includes three full years of study with an additional one year internship. SACMOs are licensed practitioners and are generally used to manage primary health care of patients.

## In each union covered by DSK, there are at least two SACMOs who have specialty training and experienced in the training of the village practitioners. Monitoring of the SACMOs will be done in accordance with the pre-existing management practice of DSK. DSK will adopt and integrate the intervention of appropriate diarrhea case management into an existing package of services.

## Sample Size Calculation and Outcome Variable(s)

## Sample size estimation for Survey

To detect a 50% increase in zinc usage in childhood diarrhoea in the intervention area, sample size was calculated using the following formula:

(Z_α/2_ +Z_β_)^2^ [p (1-p)]X 2 X C

n =

d^2^

(1.64 +1 .28) ^2^ [.15 (1-.15)]X 2 X 1.5

=

(.075) ^2^

8.53 (.1275) X 2 X 1.5

= = 580 per group

.005625

where,

n = sample size, (Z_α/2_ +Z_β_)^2^ = (1.64 + 1.28)^2^ = 8.53 (1 sided value at 95% confidence level and 90% power), p =.15 (baseline prevalence of zinc usage), d =.075 (50% increase in zinc usage after intervention), C = 1.5 (allowing an unknown cluster sampling design effect).

Given the target of 20 children per village, 29 randomly selected villages per group will be included.

# Measurement/Data collection tools

1. Household survey

Clusters (villages) will be systematically surveyed (EPI cluster survey methodology), the aim being to identify 20 children per village with an active case of childhood diarrhea until the desired sample size is obtained. Villages will be enumerated and then randomly selected. Following verbal consent, a trained interviewer will complete the questionnaire. A follow-up endline survey, also of active cases of childhood diarrhea, using the same questionnaire will be carried out six months following the baseline.

Interviewers will use a pictograph to assist parents in identifying drugs used. The study investigators will subsequently categorize them.

2. In-depth interviews with providers

## The primary aim of the interviews is to better understand diarrhoea management practices in relation to facilitating factors and important barriers to adhering with WHO/UNICEF diarrhoea management guidelines. This will include the influence of drug salesmen, financial incentives, and perceptions of what licensed providers are practicing. Assessment of these factors will be stratified by important provider characteristics, such as age, type of provider and location.

## Conduct of study and timetable

**Period: 8 Months**

| **Activities** | **Months** | | | | | | | |
| --- | --- | --- | --- | --- | --- | --- | --- | --- |
|  | **1** | **2** | **3** | **4** | **5** | **6** | **7** | **8** |
| Recruitment & Training of  Research staff |  |  |  |  |  |  |  |  |
| Training of SACMOS |  |  |  |  |  |  |  |  |
| Baseline Survey |  |  |  |  |  |  |  |  |
| Training of Private providers |  |  |  |  |  |  |  |  |
| Follow up Support |  |  |  |  |  |  |  |  |
| End line Survey |  |  |  |  |  |  |  |  |
| Analysis, write-up &  dissemination |  |  |  |  |  |  |  |  |

## Facilities Available

Describe the availability of physical facilities at the place where the study will be carried out. For clinical and laboratory-based studies, indicate the provision of hospital and other types of patient’s care facilities and adequate laboratory support. Point out the laboratory facilities and major equipment that will be required for the study. For field studies, describe the field area including its size, population, and means of communications.

ICDDR,B has a long-standing reputation of carrying out collaborative research with various national and international institutions. HSID has a good contingent of investigators, support staff, data management staff and field research staff experienced in conducting operations research. This study will be done in collaboration with Plan International and its partner NGOs, Dushtha Shasthya Kendra (DSK) and Gonoshasthya Kendra (GK). Each of these NGOs has a physical infrastructure that includes a field office, fixed and satellite service delivery points and outreach. All information regarding service providers list, coverage by service, location and total household numbers of the two catchments areas are available within these field offices. The proposed study fits well with the existing facilities of the collaborating NGOs.

## Data Safety Monitoring Plan (DSMP)

All clinical investigations (biomedical and behavioural intervention research protocols) should include the Data and Safety Monitoring Plan (DSMP) to provide the overall framework for the research protocol’s data and safety monitoring. It is not necessary that the DSMP covers all possible aspects of each elements. When designing an appropriate DSMP, the following should be kept in mind.

1. All investigations require monitoring;
2. The benefits of the investigation should outweigh the risks;
3. The monitoring plan should commensurate with risk; and
4. Monitoring should be with the size and complexity of the investigation.

Safety monitoring is defined as any process during clinical trails that involves the review of accumulated outcome data for groups of patients to determine if any treatment procedure practised should be altered or not.

Not applicable

## Data Analysis

Describe plans for data analysis. Indicate whether data will be analyzed by the investigators themselves or by other professionals. Specify what statistical software packages will be used and if the study is blinded, when the code will be opened. For clinical trials, indicate if interim data analysis will be required to monitor further progress of the study.

The investigative team will carry out the data analysis. Data entry and general management will be done by Data Resources and Surveillance Unit of the Division. Data will be entered and analyzed using SPSS version 12.0. Absolute counts, proportions, and means with 95% confidence interval will be calculated. These and regression analysis will be done using STATA version 9, cluster survey program, that accounts for potential within cluster homogeneity.

Based upon the household surveys, the following outcomes will be calculated:

- Coverage: the proportion receiving zinc, ORS (zinc and ORS), and antibiotics or antidiarrhoeals and changes in coverage 6 months following the introduction of the intervention package.
- Equity: who is receiving zinc by age, gender and socioeconomic status
- For differences in categorical outcomes crude relative risks and 95% confidence intervals will be determined.

The provider interviews will be recorded and transcripts prepared. Qualitative responses will be sub-grouped by topic and response.

## Ethical Assurance for Protection of Human Rights

Describe in the space provided the justifications for conducting this research in human subjects. If the study needs observations on sick individuals, provide sufficient reasons for using them. Indicate how subject’s rights are protected and if there is any benefit or risk to each subject of the study.

The study does not involve any invasive procedures. Caretakers of children with diarrhea will be interviewed during or shortly after an episode. In addition a selected number of providers will also be interviewed. Any child found to be moderately or severely dehydrated or experiencing bloody diarrhea will be referred to the nearest MOHFW or NGO health facility.

This study is not prescribing treatment, but promotes zinc treatment of childhood diarrhea. It will monitor trends in treatment practice, with a focus on zinc as a treatment.

Oral consent will be obtained prior to household interviews and in-depth provider interviews.

## Use of Animals

Describe in the space provided the type and species of animals that will be used in the study. Justify with reasons the use of particular animal species in the experiment and the compliance of the animal ethical guidelines for conducting the proposed procedures.

No animal will be used in this study.

## Literature Cited

Identify all cited references to published literature in the text by number in parentheses. List all cited references sequentially as they appear in the text. For unpublished references, provide complete information in the text and do not include them in the list of Literature Cited. There is no page limit for this section, however exercise judgment in assessing the “standard” length.

1. Murray C, Lopez A. Alternative projections of mortality and disability by cause 1990-2020. Global burden of Disease Study. Lancet 1997;349:1498-504.
2. Jones G, Sketetee RW, Black RE, Bhutta ZA, Morris SS and the Bellagio Child Survival Study Group. How many child deaths can we prevent this year? Lancet 2003;362:65-71.
3. Victora CG, Bryce J, Fontaine O, Monasch R. Reducing deaths from diarrhoea through oral rehydration therapy. Bull World Health Organ 2000;78:1246-55.
4. Sazawal S, Black RE, Bhan MK, Bhandari N, Sinha A, Jalla S. Zinc supplementation in young children with acute diarrhea in India. N Engl J Med 1995 Sep 28;333(13):839-44
5. Roy SK, Tomkins AM, Akramuzzaman SM, Behrens RH, Haider R, Mahalanabis D, et al. Randomised controlled trial of zinc supplementation in malnourished Bangladeshi children with acute diarrhoea. Arch Dis Child 1997 Sep;77(3):196-200.
6. Roy SK, Tomkins AM, Mahalanabis D, Akramuzzaman SM, Haider R, Behrens RH, et al. Impact of zinc supplementation on persistent diarrhoea in malnourished Bangladeshi children. Acta Paediatr 1998 Dec;87(12):1235-9.
7. Faruque AS, Mahalanabis D, Haque SS, Fuchs GJ, Habte D. Double-blind, randomized, controlled trial of zinc or vitamin A supplementation in young children with acute diarrhoea. Acta Paediatr 1999 Feb;88(2):154-60.
8. Penny ME, Peerson JM, Marin RM, Duran A, Lanata CF, Lonnerdal B, et al. Randomized, community-based trial of the effect of zinc supplementation, with and without other micronutrients, on the duration of persistent childhood diarrhea in Lima, Peru. J Pediatr 1999 Aug;135(2 Pt 1):208-17.
9. Strand TA, Chandyo RK, Bahl R, Sharma PR, Adhikari RK, Bhandari N, et al. Effectiveness and efficacy of zinc for the treatment of acute diarrhea in young children. Pediatrics 2002 May;109(5):898-903.).
10. Bhutta ZA, Black RE, Brown KH, Gardner JM, Gore S, Hidayat A, et al. Prevention of diarrhea and pneumonia by zinc supplementation in children in developing countries: pooled analysis of randomized controlled trials. Zinc Investigators' Collaborative Group. J Pediatr 1999 Dec;135(6):689-97.
11. Fontaine O. Effect of zinc supplementation on clinical course of acute diarrhoea. J Health Popul Nutr 2001 Dec;19(4):339-46).
12. Baqui AH, Black RE, El AS, Yunus M, Chakraborty J, Ahmed S, et al. Effect of zinc supplementation started during diarrhoea on morbidity and mortality in Bangladeshi children: community randomised trial. BMJ 2002 Nov 9;325(7372):1059).
13. IBLF dialogue with WHO. 2002.
14. Jones G, Steketee RW, Black RE, Bhutta ZA, Morris SS. How many child deaths can we prevent this year? Lancet 2003 Jul 5;362(9377):65-71.
15. WHO/UNICEF Joint Statement. Clinical management of acute diarrhea. WHO.FCH.CAH/04.7. 2004. Geneva, WHO.
16. Baqui AH, Black RE, El Arifeen S et al. Zinc therapy for diarrhea increased the use of oral rehydration therapy and reduced the use of antibiotics in Bangladeshi children. J Health Population Nutr. 2004;22(4):440-42.
17. Bhandari N, Mazumder S, Taneja S, Dube B, Black RE et al. A pilot test of the addition of zinc to the current case management package of diarrhea in a primary health care setting. J Pediatr Gastrenterol Nutr. 2005 Nov; 41(5):685-7.
18. Charles P Larson et al. Childhood diarrhea management practices in Bangladesh: private sector dominance and continued inequities in care. Int. J. Epidimiol. Advance access published September 22, 2006.

## Dissemination and Use of Findings

Describe explicitly the plans for disseminating the accomplished results. Describe what type of publication is anticipated: working papers, internal (institutional) publication, international publications, international conferences and agencies, workshops etc. Mention if the project is linked to the Government of the People’s Republic of Bangladesh through a training programme.

The findings will be shared with the collaborating organizations through a workshop and submission of a written report. The findings will also be presented in seminars/conferences and published in peer-reviewed journals in order to make the results available to all stakeholders. Detailed information regarding the intervention package and materials used will be made available on the SUZY Project website.

## Collaborative Arrangements

Describe briefly if this study involves any scientific, administrative, fiscal, or programmatic arrangements with other national or international organizations or individuals. Indicate the nature and extent of collaboration and include a letter of agreement between the applicant or his/her organization and the collaborating organization.

This research program intends to partner with Plan International and Dushtha Sashthya Kendra (DSK), which is a partner NGO of Plan International. DSK provides primary healthcare services along with other programs in the Gazipur district, located north of Dhaka, Bangladesh. Plan International has been working in Bangladesh to help children and families through local NGO partner to improve the lives of members of the poorest communities. Additionally, this project will relay heavily on research and materials developed during the SUZY project.

## Appendix 1

**International Centre for Diarrhoeal Disease Research, Bangladesh**

**Voluntary Consent Form (Care takers of children aged 6 months – 5 years)**

**Protocol Number:** 2007-051

**Title of the Research Protocol:** Impact of an NGO training and support intervention on private sector provider diarrhea management practices

**Principal Investigator:** Charles P. Larson

**Organization:** Health Systems and Infectious Diseases Division, ICDDR,B, Mohakhali, Dhaka 1212

**Subject ID #_____________________ Interviewer ID # _________________**

**Purpose of the research:** To assess the impact of an NGO training program developed to improve the quality of childhood diarrhoeal illness management practices among private health care providers, we have been conducting a study. This study will help us to understand how parents treat their young children when they have diarrhoea and how that might be changing as a result of provider training. Your village/community has been chosen to be part of this survey.

**Why selected:** In your village we are looking for children under five years of age who have had diarrhoea in the past two weeks. As your child had diarrhoea during last two weeks, we are inviting you to help us by participating in this study.

**What is expected from the respondent:** If you agree to participate in the study; we would ask you some questions related to diarrhoeal illness treatment of your child. Information provided by you will be of great use in designing guidelines and steps for a more successful treatment of acute childhood diarrhoea. We affirm that the information will not be used for any other purpose.

**Risks, benefits and principle of compensation:** Other than momentary inconvenience to you, we will not cause any harm to you. We should let you know that you will not be paid for participation in the study.

**Privacy, anonymity and confidentiality:** We do hereby affirm that privacy; anonymity and confidentiality of the information provided by you will strictly be maintained. Information provided by you will not be used for any other purpose than the study. You would be able to communicate freely with any investigator of this study at the address below.

**Future use of information:** Information provided by you will be a great use in designing guidelines and steps for a more successful treatment of acute childhood diarrhea in Bangladesh. However, we would like to assure you that your name would not be linked with any information that we give to others as a result of our talks with you.

**Right not to participate and withdraw:** Your participation in the study is voluntary and you are the sole authority to decide for and against participation in this study. You would also be able to withdraw your participation any time during the study. Your refusal to take part in or withdrawal from the study will involve no penalty or loss of benefits or attention. This interview will take about 1 hour or so of your time.

If you agree to our proposal of enrolling you in our study, please indicate that by putting your signature or your left thumb impression at the specified space below.

Thank you for your cooperation.

_________________________________ Date:

Signature or left thumb impression

[

_________________________________

Signature of the PI or his/her representative Date:

**Budget**
